# The why, what and how of preconception care: an exploratory descriptive qualitative study in Karnataka, India

**DOI:** 10.1186/s13690-023-01180-6

**Published:** 2023-09-29

**Authors:** Agnita Robert Narendra, Ambuja Kowlgi, Gururaj H Patil, Swaroop N, Arin Kar

**Affiliations:** Maternal, Neonatal, Child Health and Nutrition Thematic, Karnataka Health Promotion Trust (KHPT), Bengaluru, India

**Keywords:** Preconception care, Pregnancy outcomes, Socio-cultural, Care-seeking behaviour

## Abstract

**Background:**

Women’s health and nutrition are key to their reproductive health and are important for optimising pregnancy outcomes. Formation of most foetal organs starts soon after conception and much before the woman has her first antenatal visit. The provision of biomedical, behavioural and social interventions to couples to address health, nutrition, behaviour issues and individual environmental risk factors that could contribute to improved maternal and child health outcomes before conception is crucial. Most rural women in India, do not seek pregnancy care before the second trimester because of socio-cultural factors. Therefore, intervening in the preconception period is important. The objective of the study was to explore the challenges and opportunities of implementing preconception care interventions.

**Methods:**

Individual, in-depth, semi-structured interviews (n = 25) were conducted with primary stakeholders (newly married women, newly married men, and family members) in Shorapur taluk of Yadgir district and Devadurga taluk of Raichur district. Thirty-one interviews were conducted with taluk, district, state officials and academicians. This descriptive qualitative study conducted four focus group discussions with front-line health workers. The in-depth interviews (IDIs) and Focus-group discussions (FGDs) used separate pre-tested semi-structured interview/discussion guides. Data analysis was carried out using NVivo software using a phenomenological approach with both inductive and deductive analysis.

**Results:**

A strong influence of social and cultural norms shapes healthcare-seeking behaviour at the community level. Poor dietary diversity, lack of awareness, poor literacy levels, work pressure for women, lack of decision-making power and empowerment among women, pressure to conceive early, and gender norms are the roadblocks to successful preconception care programs in the rural Karnataka setting. The stakeholders expressed the need for interventions during the preconception period. The government functionaries recommended several interventions which could be potentially integrated into the existing Reproductive Maternal, Neonatal, Child and Adolescent Health (RMNCH + A) strategy to improve the health and nutrition of women before they conceive.

**Conclusion:**

The study highlights the need for structured interventions during the preconception period to improve maternal health and pregnancy outcomes. The recommendations provided by government functionaries are indicative of the feasibility of integrating interventions in the RMNCH + A strategy.

**Supplementary Information:**

The online version contains supplementary material available at 10.1186/s13690-023-01180-6.

**Table:** 

**Text box 1.** Contributions to the literature
• The need to start care provision during the preconception period is very evident due to certain cultural norms in the rural setting in Karnataka including early marriage, pressure and expectation to conceive early, late disclosure of pregnancy and delayed care-seeking behaviour among women and their families.
• The concept of preconception care generally resonated and was supported by all government officials and healthcare providers, but requires targeted interventions in the care continuum within the existing Reproductive Maternal, Neonatal, Child and Adolescent Health (RMNCH+A) strategy in India.
• The qualitative study findings provide a basket of interventions suggested by government functionaries which include leveraging existing programs and new interventions during the preconception period targeted at women of reproductive age.
• The state governments can translate the recommendations from this study into a realistic plan for effective program implementation and monitoring.

## Background

Maternal and child mortality have considerably decreased in India during the past decade [1, 2]. The state of Karnataka has likewise experienced a downward trend [3]. Despite the progress, the country has an uphill task of achieving the Sustainable Development Goals for 2030. Morbidities including anaemia, undernutrition, overnutrition, hypothyroidism, infections (sexually transmitted/reproductive tract) and non-communicable diseases (diabetes, hypertension) increase the risk of adverse birth outcomes in women of reproductive age. Maternal healthcare is one of the cost-effective factors for the prevention of unfavourable birth outcomes [4].

The National Health Mission’s Reproductive Maternal, Neonatal, Child and Adolescent Health (RMNCH + A) strategy in India focuses on adolescent, maternal and child health [5]. The preconception period remains a neglected phase in the continuum of care. Preconception care is defined as “any preventive, promotive or curative health care interventions provided to women of childbearing age in the period before and between consecutive pregnancies to improve health-related outcomes for women, newborns and children up to 5 years of age”. It is suggested that interventions during pregnancy can be more effective if initiated before conception [6]. Most foetal organ development begins shortly after conception, well in advance of the woman’s first prenatal visit [7]. Early risk assessment and interventions, such as preconception care, are essential for women of reproductive age, as they help improve mother and child health outcomes [8]. The risk factors, behavioural issues and health problems can be effectively addressed before conception through biomedical, behavioural and social interventions [8].

Global evidence suggests that preconception care interventions are effective in improving mother and child mortality and they can be integrated into the maternal and child health package [7–10]. In India, extending the RMNCH + A interventions to the preconception period is still a possibility. At the state and national levels, governments will need to adopt an informed strategy to devise a sustainable preconception care program. It is imperative to first assess the situation, narrow down the interventions that should be prioritised, decide who should receive them, and specify the delivery method.

An improved understanding of various stakeholders’ knowledge, attitudes and behaviours is essential to formulate targeted interventions and effective implementation strategies during the preconception period. To answer this, KHPT conducted a qualitative study in Yadgir and Raichur districts using a qualitative descriptive exploratory design [11] to understand the challenges and opportunities of preconception care. The study also aimed to identify a package of social and health interventions to be delivered to eligible couples and their families through RMNCH + A services.

## Methods

The study used a qualitative descriptive approach to understand the opportunities and challenges to integrating preconception care in the RMNCH + A strategy from various stakeholders. Ethical approval for this study was obtained from the Institutional Ethics Committee of M S Ramaiah Institute of Applied Sciences, Bangalore. Written informed consent was obtained from all participants before the study. Study participants fell into three categories: (1) **primary stakeholders**, including the newly married women (NMW), newly married men (NMM), and family members, (2) **secondary stakeholders**, including community, taluk, district, and state officials; and (3) **tertiary stakeholders**, including academicians and other organizations.

### Recruitment

Raichur and Yadgir were selected as they are in the category of aspirational districts initiative by the 101 Government of India [12]. Within these two districts, Devadurga in Raichur and Shorapur in Yadgir were selected for qualitative research. We followed a stratified two-stage approach for recruiting primary stakeholders. In the first stage, villages were selected (simple random sampling) in the taluks Shorapur and Devadurga. In the second stage, below-poverty-line households were selected from the randomly selected villages. A frontline worker- Accredited Social Health Activist (ASHA) from the selected villages shared the list of newly married couples. Participants were then randomly recruited from this list for in-depth interviews.

### Data collection

The study used focus group discussions (FGDs) and in-depth Interviews (IDIs) for data collection. The study was conducted between September 2021 through January 2022. Focus group discussions (FGDs) with frontline workers were conducted using semi-structured guides. The IDIs and FGDs used separate pre-tested semi-structured interview/discussion guides. The questions in the guide focused on the perspectives of stakeholders about the need for preconception care, barriers and facilitators to the preconception period and recommendations to integrate preconception care into the existing strategy. Demographic information was collected from the primary stakeholders at the beginning of the interview. All the interviews and focus group discussions were audio-recorded, translated, and transcribed verbatim and the transcripts were reviewed for accuracy.

### Data analysis

Data was translated from Kannada to English, the transcripts were then coded. Data was cleaned and filtered; the codes were transferred to a matrix and the broader emerging themes were charted. Deductive coding has been used to organize data into the data matrix. Further, the data has been sorted into three broad categories that are relevant to the study purpose (challenges, opportunities and recommendations). Inductive analysis has been used to analyze the data collected through interviews and focus group discussions. Open coding has been done by the research team reading through the data and developing and applying codes to represent voices in the data. The patterns emerging have been revisited by the research team to reduce bias. The data presented in the results in the following section includes perspectives of the stakeholders as well as the interpretation from the consolidated analysis. The voices of the stakeholders are presented as quotes in italics and are retained in the verbatim format. The qualitative data analysis software NVivo was used for documentation and organizing data into themes and sub-themes.

## Results

A total of ten newly married women and 7 newly married men consented to participate in in-depth interviews which lasted an average of about 60 min each. Data saturation guided the decision for the sample. The interviewed newly married women constituted a relatively homogenous group demographically, within a narrow age range of 18–23 years. There was an even split between endogamous and exogamous marriages (defined as marriages within and outside of the family, respectively). Compared to the NMW, the NMM had a wider and older age range of 21–32 years. About 5 men were college-educated, with one out the seven being illiterate and the remaining two having primary school education. A much greater proportion of the NMM (all except one) was in endogamous marriages. Characteristics of the participants are summarized in Table 1.

**Table 1:** Characteristics of the participants (primary stakeholders) interviewed in taluks Shorapur and Devadurga between September 2021-January 2022

Categories		NMW (n = 10)	NMM (n = 7)	Family (n = 8)
Age range		18–23	21–32	40–60
Gender	Male	NA	NA	4
	Female	NA	NA	4
Religion	Hindu	9	6	7
	Muslim	1	1	1
Highest formal education	1-7th Grade	2	0	1
	8-10th Grade	2	1	0
	Pre-University	2	0	1
	Graduation	3	3	0
	Professional course	1	2	0
	Illiterate	0	1	6
Caste	OBC	5	5	5
	SC	4	0	2
	ST	1	2	1
Type of marriage	Endogamous*	5	6	7
	Exogamous**	5	1	1

* Endogamous marriage is marriage within a specific group as required by custom or law

** Exogamous marriage is marriage outside one’s group

OBC, Other Backward Caste; ST, Scheduled Tribe; SC, Scheduled caste

Additionally, 31 In-depth interviews (IDIs) of secondary and tertiary stakeholders along with Four focus group discussions (FGDs) with frontline workers were conducted.

Four main themes emerged from the data: (1) Why is there a need for preconception care? (2) Stakeholder’s perceptions and awareness about the need for preconception care (3) What interventions to deliver before conception? (4) How to deliver preconception care interventions?

### Why is there a need for preconception care?

There is enough scientific evidence to establish the need for preconception care. In the given rural north Karnataka setting, there are several factors that this qualitative study provides to emphasise the need for such care for women of reproductive age. The focus group discussions and interviews have shed light on the many obstacles that women face while trying to access healthcare services. Access to preconception care services, when available, may also be hampered by these challenges. The elements listed below highlight the necessity of care before conception.

#### Pressure to conceive early and unplanned pregnancies

All the stakeholders mentioned the social expectation of early conception. Many recognised that it is often the first question asked in social interactions once the couple is married. The married woman is expected to bear a child within the first year of marriage.

Significant proportions of the primary and secondary stakeholders plainly stated that in their experience, couples generally try to conceive following marriage. Interestingly, the newly married male interviewees did not bring up the topic of societal expectations around conception, and many of them shared that they thought of conception in terms of ‘destiny’, and something that’s up to divine intervention. None of them talked about reproduction as something that they can control or influence through contraception and consultation with healthcare professionals.

*As far as I have seen, it is like you said, they say we get it when God gives. (NMM01RD, NMM).*

The healthcare providers and government functionaries, consistently raised stress as a serious health risk facing newly married women—one that has real physical manifestations that may intersect with dietary behaviours as well as general maternal health and birth outcomes.

*After three months, if she is not pregnant kirukula (torturing) begins… No one will like her, even husband will not like her. They were saying to him, what is this, it’s been six months and your wife has not given any news, it (periods) has not stopped? (HCP01RD, RKSK counsellor).*

#### Lack of self-care among women

Due to the predominant gender norms in Northern Karnataka, women have a socially prescribed role in households as the primary caretakers. They are expected to always be vigilant and mindful of other family member’s health, but this concern is not always reciprocated by men in the household, leaving women in a more vulnerable position. A panchayat development officer in Raichur points out, this dynamic can also exacerbate public health risks when the women who are tasked with safeguarding their family’s health are not sufficiently equipped with the information and resources needed to ensure both they and their household eats healthy food. Similar thought was also shared by another stakeholder:

*…what they (women) do, they cook vegetables and give them to their husbands, children, and mother-in-law, serve everyone and after feeding everyone, if anything is remaining, they eat… pregnant women also are not aware of their nutrition. They think that their work is to serve their husband and children, for them, it is their biggest duty. (DDWCDYS)*

As a concrete example of how prioritizing others’ health can come at a cost to women’s health, some respondents mentioned that women who have large families may accept, cook, and serve government-supplied provisions to family members—but because they eat last, the women don’t get enough of the nutrition for themselves. A newly married woman’s statement below is a testament to the same:

*First men eat and then we do. Like that only, they eat first and then we eat. It is paddhathi (tradition). I don’t feel anything (about this), how can I (we) eat unless they have eaten, so I don’t eat (till they finish eating). (NMW02RD, NMW)*

As a related issue, whether it’s due to restriction of healthcare-seeking by husbands or elders, a lack of awareness of the importance of ANC services, or simply prioritizing others’ health at the expense of their own.

#### Work pressure for women

The stakeholders pointed out that although women work in agricultural fields, their household responsibilities have not necessarily diminished, as they are still expected to manage housework such as cooking, and care for family members. Their packed schedules interfere with their nutritional intake given that they are more likely to cook what is easiest, as opposed to what is healthiest. For example, they might cook foods that they’ve been accustomed to making from an early age, like roti (Indian flatbread) and daal (lentils). When not balanced with sufficient quantities of fresh produce and iron-rich foods, the result is malnutrition.

They have cookers now, anna, byali they will cook in the cooker and leave, they go to work in the field. Till night also if they eat anna byali only what they will get, Government easily gives rice, byali is available, they easily prepare… There is also pressure on them, to go, to work and earn, more wages are there now, want to go so they find easy thing. (MORD)

Respondents across all participant groups remarked on women’s taxing work routine. Multiple informants, including a NMM, mentioned that women’s demanding work pressures do not let up even during and directly after pregnancy:

*Women compulsorily work in the village; they do all the work in the village. Pregnant women work up to the eighth month. (NMM06YS, NMM)*

*…women go to work in the morning at 8.30 or so and don’t come home till 7.00 in the evening. See since this irrigation started, it is like this (AWWRD)*

*…in the fields, only they work. After 10.00 AM, no one is available in the village at all… NMW also work, no one will be there in the house. They go… Even after delivery, they don’t stay back for a month also… In some houses, if they go in the morning, they will come back in the evening at 6.00 PM. They carry a lunch box with them and go, they take it for the afternoon. (PHCORD)*

#### Lack of decision-making powers of women

Reportedly, decision-making power as well as the freedom to speak and voice one’s opinion increases with the birth of the first and second children. The newly married women interviewed reported that the decision of the first conception doesn’t lie in their hands. However, it is also pointed out that by the time she reaches the phase in life when she can voice her concerns, it makes no difference because by then, societal pressure to conceive will have reduced but whatever impact was to happen on her health would have already happened.

*I will only decide about the gap, I am the husband, I will only decide about that, my wife will not decide about that. (NMM04YS, NMM)*

The decisions around the purchase of food items are taken by the husband or the in-laws and the newly married woman is not involved in the decision-making process. This can affect the intake of nutritious food by the newly married woman.

*Vegetables and all my mother-in-law will only bring, and elders in the family will only bring. Maybe my father-in-law or mother-in-law will bring it, not me. Elders whoever will be free will do all those things. (NMW04RD, NMW)*

#### Adjustments with new family post-marriage

Typically, newly married women move into their in-law’s houses, which requires significant adaptation, and this can be a stressful period. There may be different meal schedules, dietary preferences, and general life patterns amongst the members of the new household, as compared to the girl’s natal household. Newly married women often pointed out that there are adjustments to be made; there is also anxiety and hesitation to speak with in-laws. Compared to in-laws’ house there is more freedom in the natal house. Freedom concerning mobility and involvement in family issues at different levels increases after the birth of the first child.

Both consanguineous and non-consanguineous marriages are common in the study areas. There exist mixed opinions about the advantages and disadvantages of each. However, as data suggests, girls who marry outside of their kin have greater difficulties adjusting to a new home.

*There is mujugara (hesitation) because it is a new house, new place, new people…in all ways, in eating, in doing work. There is a little mujugara like this. What work they will do, what work we should do, I didn’t understand, now I understand and I do everything. Now it does not feel like that (NMW10YS, NMW).*

Some of the newly married women expressed that they are hesitant to speak with their spouses. This is also mentioned by other interview participants. Often it is mentioned that the newly married feel scared to speak their mind to their mother-in-law or father-in-law, as the healthcare provider says:

*The newly married woman may not even drink water, if the father-in-law is sitting near the water container, she may not drink water for 2–3 hours if he sitting there for that long. (CHO06YS, MLHP).*

*There will be a lot of hesitation. I am eating more or what, they will be watching, what they will think… they may say, she eats this much rotti (flat bread), she eats this much rice, she thinks like that. But it is not like that in the mother’s house. (ASHARD)*

The adjustment process can be hindering the self-care and dietary habits which is crucial for the good health and nutrition status of a woman. Compromised health and nutrition status before conception can pose a risk factor for healthy motherhood and birth outcomes. Generally, the conception takes place in this phase where the woman is still adopting the new status of being a daughter-in-law and adjusting to the norms and practices of the household, as such it adds to the vulnerability.

### Perceptions and awareness about preconception care

The concept of preconception care is novel for the community. The stakeholders pointed to the fact that there are no interventions currently being implemented that target women prior to conception and all the existing interventions focus on women once she conceives:

*…we don’t have that program (newly married couples) …But I don’t have an idea about that. Only after pregnancy we enrol, after delivery, we enrol those who have anyone come from outside. (CDPORD)*

*There is no scope for that (including newly married in VHND and meetings for pregnant women), that category is not there, they have not been included in any of these things…. We are also neglecting them, where there are injections for them? where there is a provision to call them? after they become pregnant, we start check-ups for them. there is no program to involve them in between. (CDPORD)*

* I don’t know about this, I don’t have information about this (NMM04YS, NMM)*

As part of the study, we tried to understand the views around introducing preconception care services from all the stakeholders. Many, although not all, of the stakeholders, revealed their stance towards the idea of strengthening and expanding programs to improve women’s nutrition at the preconception stage. The Family and Community Members group was almost evenly split between those who overtly voiced support for intervening further upstream, versus those who either did not speak to this topic or voiced pessimism about such an approach. Three out of the seven NMMs had a positive opinion, and seven out of 10 of the NMWs were supportive of preconception care programs. The responses have been mentioned below:

*Yes, it is necessary that she has to take care of it… No, we have not done any preparations. (NMM07YS, NMM).*

*Information should be given in the school and college.… if a demonstration is given it will be good, it will be helpful. (NMW06YS, NMW)*

*There is no need to bring (anything and give) before pregnancy. (NMM06YS, NMM)*

The public healthcare providers and policymakers expressed support for preconception nutrition efforts. The District Officials group was generally supportive of preconception nutrition awareness-raising, with only one-third not voicing support. The recommendations from government officials and policy implementers highlighted the need for such interventions in improving maternal and child health outcomes:

*We are doing it for pregnant women, we can do it for them also (newly married women). Then they will look after correctly…we are doing so much work, along with that we can also tell a few things to the newly married women. (AWWRD)*

*…reaching her before (pregnancy) is important because, if she knows all the information, if she gets all the information, then, in future when she becomes pregnant, she will know how she should be and how she should eat, all this information she will come to know, she will tell her family about that also. (AWWRD)*

Much difference of opinion was obtained from stakeholders for the appropriate timing for targeted awareness-raising and nutrition or health interventions. Stakeholders fell into one of three groups: those who advocated for intervening only after pregnancy, those who preferred targeting all newly married women, and those who believe that waiting till marriage to raise awareness is too late.

*That we can think after becoming pregnant. (NMW01RD, NMW)*

*…to get that outcome or prevent anaemic pregnancy or maternal death, infant death all those things. So we have to concentrate more on adolescents (MORD)*

Stakeholders mentioned that eligible couple listing is a routine exercise by ASHAs and there is an opportunity to integrate interventions identified exclusively for the couples before pregnancy along with family counselling:

*The eligible couples (EC) listing is happening continuously. (MORD)*

The data from our qualitative research emphasised that leveraging key services and programs in RMNCH+A could act as a prime facilitator for preconception care. During in-depth interviews and FGDs, participants provided insights on the what and how of the interventions for the preconception period. The state and district-level officials and healthcare providers offered programmatic recommendations, given their professional focus, on facilitators for preconception care; many of these were echoed and supplemented by the rest of the participant groups.

### What interventions to deliver during the preconception period?

The participants proposed areas of intervention, which can work towards addressing the barriers listed above and provide adequate care to women before conception. They fall under the following broad themes and have been summarised in Table 2:

1. Leveraging existing programs.
2. New interventions.

#### Leveraging existing programs

The existing programs and schemes by the state and central government are comprehensive to deliver various maternal, antenatal and postnatal interventions and to reduce maternal and child mortality. Most of the maternal health programs focus on women once she conceives and these interventions can be leveraged to support women before conception.

The suggestions mentioned below can help optimise the existing interventions to aid in improving care during the preconception period:

• **Nutrition-related interventions**

Iron and folic acid tablets are provided to pregnant women after registration and the opinion of a few stakeholders was to initiate the supplements before a woman conceives to help her have good stores before she conceives:

*…Provide nutrition supplementation for those who need it. Expand RBSK / RKSK programs to include these components. (SNO).*

*…Then, tablets and all, calcium tablets, they can’t buy, so the government gives calcium tablets. Whatever they need those should be supplied. (NMWYS07, NMW)*

The provision of food in the form of hot cooked meals was strongly recommended to bridge the gap in nutrient intake. Stakeholders thought to extend the provision of hot cooked meals as part of the Matrupoorna Yojane by the government of Karnataka which is presently provided to pregnant women to undernourished women before conception:

*They should give them meal there only because they give ration and all, and if they give it home, I don’t know if they will get it (use it for themselves or not) or not. They should call them there, give them food there and tell them, eat egg and all like this… they prepare it for the children and give (NMWRD01, NMW)*

*…They should give nutrition food to those whose nutritional status is not good, like they give eggs to pregnant women, like that they should give it would be best. (NMWYS06, NMW)*

*…There should be some service to give them some nutrition or something after marriage may be for three to six months… may be some powder for six months after marriage… After that, if we want, we can continue. Or we can just complete six months course and if they are pregnant, we can continue with other things. (MLHP01YS)*

• **Education and Awareness-raising**

There was greater unanimity on this theme than any other; every stakeholder group repeatedly emphasized the need to better educate the public regarding nutrition. A few stakeholders recommended a robust awareness-raising campaign:

*A concerted communications/education campaign needs to be rolled out to ingrain the importance of nutrition and raise awareness of things like haemoglobin. (SNO)*

Other interventions proposed included expanding nutrition education in schools:

*Nutrition education in school should be improved/expanded. Starting in 4th standard, kids should be taught about healthy dietary habits, vitamin deficiencies and diet-related illnesses. Currently, private schools teach this but not public-sector schools. (SNO)*

Others suggested educating parents in particular:

*Parents should have the knowledge my daughter is growing and she needs more nutrition and extra education about nutrition for future needs. So those kinds of things are there. knowledge level should improve then everything will improve. Especially from the school level we should start explaining, that is very much needed. By the time she comes to a doctor, she will be anaemic. She will be anaemic, she will be malnourished, and she will be underweight. So, if we start from basic it will be good. (RCHOYS)*

Besides education at the family level, the Healthcare Providers (HCPs) should provide adequate messaging and information about the services provided to the beneficiaries and their families for effective utilization and impact. For example, there is room for improvement in ASHA workers’ messaging and counselling, when instructing pregnant women on the proper use of supplements:

*ASHA workers have been instructed to go house-to-house and ask pregnant women to ingest supplement tablets on the spot. If this isn’t done, the pregnant women will often decline to take them due to aversion to black stool. ASHAs need to explain that this is normal and isn’t a cause for concern. And emphasize the importance of maintaining a healthy level of haemoglobin. (CEOYS)*

**New interventions**

One new intervention that can be included along with leveraging existing intervention is screening and monitoring the health and nutrition status of the newly married women. Specific recommendations were made, such as screening and monitoring of newly married women to ensure a woman enters pregnancy with good health and nutrition status:

*Screen prospective mothers for any other disorders (non-nutrition related) that could cause co-morbidity; assess mental health; assess the availability of cheap local nutritious food; screen for reproductive tract infections; provide nutrition supplementation for those who need it; expand Rashtriya Bal Swasthya Karyakram (RBSK)/ Rashtriya Kishore Swasthya Karyakram (RKSK) programs to include these components and improve coordination across existing programs. (SNO)*

*Emphasise on measurement… Haemoglobin needs to be quantified by using testing strips at the point of care. Protein can be similarly tested. Women need to be screened for underlying conditions and co-morbidities and categorized accordingly. Newly married women must be put on a tailored therapeutic course based on what their specific health needs are. The injection might be more effective than supplement tablets for those with more acute deficiencies. (Academician)*

The recommendations were broadly to screen women for health and nutrition indicators particularly, anaemia, body mass index, Diabetes Mellitus, hypertension, thyroid disorders and mental health.

**Table 2:** Summary of the suggested interventions during the preconception period by different stakeholder groups who were interviewed between September 2021-January 2022

Leveraging and strengthening existing programs to include care during the preconception period	
Existing programs	Inclusion
**Poshan Abhiyaan Program** [13]	Poshan Abhiyaan can be extended to provide nutrition education and reproductive health counselling awareness before pregnancy through: 1. Counselling initiatives for newly married couples and awareness for couples and families through Primary Health Centre teams (including Health and Wellness Centres) and frontline workers 2. Awareness programs for other key community stakeholders such as Gram Panchayat members and religious leaders 3. Awareness programs for adolescents on preconception care, which can be a multi-department effort
**Village Health, Sanitation and Nutrition Committee (VHSNC) and Village Health and Nutrition** **Day (VHND)**	VHSNC and VHND meetings can be leveraged for preconception care discussions
**Pradhan Mantri Surakshit Matritva Abhiyan (PMSMA)** [14]	The PMSMA camps, which are scheduled every month, can include a preconception care concept
**Anaemia Mukt Bharat program under the National Health Mission** [15]	Micronutrient supplementation of iron and folic acid provided as part of the anemia mukt Bharat program for women of reproductive age can be ensured for women planning to conceive
**Mathrupoorna Scheme** [16]	The hot cooked meal provided to pregnant and lactating women through the existing system of Anganwadi Centers as part of the Mathrupoorna Scheme can be extended to undernourished women planning to conceive.
**Potential new interventions targeting preconception nutrition care planning**	
**New interventions**	**Activity**
**Screening & Management**	1. Calculating Body Mass Index (BMI) 2. Hb estimation for anaemia prevention and treatment 3. Testing for diabetes, thyroid disorders, hypertension other biochemical estimations 4. Regular follow-up - health check-ups for weight and underlying condition management

### How to deliver preconception care services?

The secondary and tertiary stakeholders have suggested how preconception care services can be provided to newly married couples. They include:

• **Involving the gram panchayat to deliver preconception care services**

The predominant strategy to deliver preconception care services suggested by stakeholders was to involve the whole panchayat in counselling newly married couples at the community level:

*In this season (marriage season, February-May) we can have these awareness programs, we can invite them and like this… what we should do is, at the panchayat level, we should call the newly married couple and we can give them training. (EOYS)*

Involving the Gram Panchayat is the way forward to ensure good health and nutrition of NMW, as suggested below:

*At the panchayat or PHC level, kitchen garden or other diet-focused interventions should be rolled out. (MORD)*

*…what we should do is, at the panchayat level, we should call the newly married couple and we can give them training… If we hand this over to the village health committee, it will be at the village level and it will not be a burden. I suggested gram panchayat, but because there is this team at the village level, they need not come to the village panchayat level also. (EOYS)*.

*…as PDO I can bring together the community and other departments. Gram panchayat is there to bring all departments together and carry on the activities. So, in that direction as PDO I can bring together people and whatever committees are there, I can support them to achieve the objectives and also deal with malnutrition. Like that, we should take precautions. Before pregnancy, if we take precautions then if there is any health issue like thyroid, it can easily be managed. (PDOYS)*.

Skill development courses for adolescent girls at the panchayat level were suggested for life skill development about food, cooking, nutrition, and family management.

**• Engaging the husband and family members of the newly married women**

Another strategy suggested was the engagement of men and family members in decisions around the health and nutrition of the women. Participants of the study emphasized educating various members of the household on the importance of not only nutrition but generally caring for and ensuring the health of newly married women.

*… also, elders should be given this information, they will also help. Parents will be there in that house, mother-in-law, father-in-law, they only look after us, isn’t it? So, they should be given information. in meetings also they can tell, and they can tell during house visits also. (NMW04RD, NMW)*

Educating men and creating awareness on nutrition and birth spacing and making them aware that women’s health should be of high priority as suggested below:

*“Main role should be men, we should give awareness to men only, and we should say, see these women have come trusting you… She gives birth, you should care about her, show concern for her, you should feed her, and show affection. We should tell them; we should tell them only.” - (PDORD)*

Numerous responses from NMM like the one below made clear that such engagement has been lacking, and that healthcare providers and educators have failed to reach out to them with relevant information and guidance.

*“No one has spoken about this with me. No one has told me about this. No information, no one has told. I have not spoken to anyone about this” - (NMM02RD, NMM).*

• **Convergence and collaboration across different government departments**

Strengthening intra- and inter-governmental coordination, both laterally (across departments) and vertically (national, state, and district levels) was suggested by stakeholders for the effective implementation of preconception care programs. There is an inclination towards collaborative efforts rather than ownership by a single department:

*Mainly school, education department, anganwadi, health, Integrated Child Development Services (ICDS) and health. Three of them should combine. (MORD)*

**• Clear articulation of respective roles and responsibilities of health cadres for the provision of preconception care services:**

*For successful implementation, the three ‘A’s need to be involved: ASHAs, Auxiliary Nurse Midwife (ANM), and Anganwadi. ASHAs will be mobilizers, ANMs will be health providers, and Anganwadi will be nutrition advisors. In case communities resist the three ‘A’s (ASHA, anganwadi worker (AWW) and auxiliary nurse midwife (ANM)), village leadership should be engaged.” (SNO)*

• **Encouraging greater autonomy and leeway for customization of preconception care interventions at the district level (using Needs Assessments):**

*“Each district has its unique context, and they should be able to determine which specific age range should fall in the “preconception” period. The pubertal period is critical for nutrition as that’s when the growth spurt happens.” (Academician)*

*Community Needs Assessment (CNA) can help understand specific needs. It is being re-started in the context of Comprehensive Primary Healthcare (CPHC). (Academician)*

*National and state levels need to be very involved at the planning stage but districts need to be given enough autonomy to innovate and develop their own tailored approaches. Expectations around data reporting need to be reasonable so as not to overly burden the implementation process. (Academician)*

## Discussion

The study findings presented distil the perspectives and insights of relevant and diverse stakeholder groups including policy implementers, healthcare providers, family members, and newly married couples. The research team has endeavoured to contribute to the evidence base on this topic and compiled strategies recommended by stakeholders for integrating preconception care in the care continuum.

Although obstetric causes might seem the most evident factor leading to poor birth outcomes, it is the tip of the iceberg and there are often multiple and complex interconnected issues driving the problem. There is a need to shift the perspective and look beyond the visible issues. Preconception care is one such aspect that directly affects maternal and child health outcomes yet goes unnoticed. It is imperative to consider preconception as a crucial phase in the care continuum to see improved outcomes.

The stakeholder interviews and discussions revealed that the concept of preconception care generally resonated and was supported by all government officials and healthcare providers, but few of the primary stakeholders questioned the need and were of the mindset that no type of preparation for pregnancy is necessary. One key disagreement point among many stakeholders was around the appropriate timing for introducing preconception care interventions. Stakeholders fell into one of three groups: those who advocated for intervening only after pregnancy, those who preferred targeting all newly married women, and those who believed that waiting till marriage to raise awareness is too late. However, the South East Asian expert group consultation suggested that preconception care should be introduced with a dual focus: one on reducing maternal and newborn morbidity and mortality and the other on enabling adolescents to make a healthy transition into adult life [8].

The need to start care provision during the preconception period is very evident due to certain cultural norms in the rural setting including early marriage, pressure to conceive early, late disclosure of pregnancy and delayed care-seeking behaviour among women and their families. Some of these barriers were reported in other studies identifying factors influencing the provision of care during the preconception period [17, 18].

Further, the notions people and communities carry around life phases and associated behaviours determine access to care services. For example, healthcare providers interviewed mentioned that pregnancy is conceptualized as a natural state by rural communities and that it does not require much attention. Public awareness can be created over a period of time and requires intensive efforts to build on the beliefs of the communities. Addressing the barriers to care including the socio-cultural barriers may have a greater likelihood of success in the uptake of care services and particularly during the preconception period. The culturally competent care program for Australian Aboriginal women is an example where culturally appropriate care provided by healthcare providers led to an increase in the uptake of maternity services [19]. Empowerment though is a long-term solution that needs to be addressed concerning change in attitudes, knowledge, understanding the importance of self-care and creating opportunities to access care.

This qualitative study is a compilation of recommendations from the healthcare providers at the taluk and district level together with state and district level government functionaries about interventions during the preconception phase of the care continuum. The recommendations ranged from expanding existing interventions to newly married couples to new initiatives within the ambit of RMNCH + A. The interventions can be categorised into the following domains: education and awareness, nutrition-related interventions, health and nutrition screening with monitoring and family-focused counselling. Most of the domains listed here align with the preconception intervention packages laid out by Lassi et al. which focus on five packages of interventions. They include screening and management of chronic diseases, nutritional counselling and family planning, nutritional optimization, continuing secondary education for adolescents and multicomponent youth development programs [20]. Further, the various recommendations provided by government functionaries are part of the preconception care programs of other countries [21, 22].

The government will need to translate the recommendations into a realistic plan and costing for effective program implementation and monitoring. A multi-sectoral comprehensive approach in the interventions is key to improving a woman’s health and nutrition status before she conceives and thereby better pregnancy outcomes. The achievement of the SGD goals depends on efficient coordination and collaborative efforts across ministries and stakeholders.

The study is limited by its geographical representation and the qualitative nature. As an extension to the outcome of this study, implementation research, demonstration projects and experimental studies can be undertaken to understand the delivery strategies for preconception care interventions and their feasibility in terms of acceptability, coverage and cost. Leveraging existing programs within the RMNCH + A ambit might accelerate success.

## Conclusion

The qualitative study outlined the various challenges and opportunities for preconception care. There is a consensus on the need for extending the interventions to the preconception period. This study highlights the need for fostering a culture where preparing for pregnancy is a way of life. Structured preconception interventions set the path for improved mother and child health, lowering morbidity and mortality rates.

### Electronic supplementary material

Below is the link to the electronic supplementary material.

Supplementary Material 1

Supplementary Material 2

## Data Availability

The dataset used and analyzed during the current study is available from the corresponding author.

## List of abbreviations

ANM: Auxiliary Nurse Midwife
ASHA: Accredited Social Health Activist
AWW: Anganwadi Worker
BMI: Body Mass Index
CNA: Community Needs Assessment
CPHC: Comprehensive Primary Health Care
FGD: Focus Group Discussion
HCP: Healthcare Provider
ICDS: Integrated Child Development Services
IDI: In-depth Interview
KHPT: Karnataka Health Promotion Trust
NMM: Newly Married Men
NMW: Newly Married Women
RBSK: Rashtriya Bal Swasthya Karyakram
RKSK: Rashtriya Kishor Swasthya Karyakram
RMNCH + A: Reproductive, Maternal, Newborn, Child & Adolescent Health
PHCO: Primary Health Care Officer
VHSNC: Village Health Sanitation and Nutrition Committee
VHND: Village Health Sanitation and Nutrition Day
