# Supplementary material for: The why, what and how of preconception care: an exploratory descriptive qualitative study in Karnataka, India
Source: Arch Public Health. 2023 Sep 29;81:177. doi: 10.1186/s13690-023-01180-6 (PMC10540457; doi:10.1186/s13690-023-01180-6)
Supplement: Supplementary file 1 — Supplementary Material 1 [file 13690_2023_1180_MOESM1_ESM.pdf]

1. **Query: Why these two taluks?**

Response: These districts were selected based on socio-economic and health outcome indicators. Within these two districts, one taluk was selected for qualitative research in each district: Deodurga in Raichur and Shorapur

in Yadgir. The Government of India has launched the 'Transformation of Aspirational Districts' initiative in January 2018 with a vision of a New India by 2022, where the focus is to improve India's ranking on the Human

Development Index, to raise living standards of its citizens and ensure the inclusive growth of all. Raichur and Yadgir were selected as they are in the category of aspirational districts.

Change in manuscript:

Raichur and Yadgir were selected as they are in the category of aspirational districts initiative by the Government of India [12]. Within these two districts, Devadurga in Raichur and Shorapur in Yadgir were selected for qualitative research.

Page no. 5; line no. 100-102

2. **Query:** Why? Justification?

Response: Maternal and child health care and nutrition indicators are worse among the below-poverty-line households than in their non-poor counterparts. The levels of antenatal care, safe delivery and childhood vaccinations are much lower among socioeconomically disadvantaged households.

3. **Query:** How many?

Response: 02 FGDs with ASHAs and 02 FGDs with AWW

Change in manuscript:

The study used focus group discussions (FGDs) and in-depth Interviews (IDIs) for data collection.

Page no. 5; line no. 109

4. **Query:** How many?

Response: Details are in the results section

5. **Query:** From the eligibile couples' list what was the strategy to select women/ men-? Why the number of 10 and 7? Were they couples? The sample of family was a different set? Who were interviewed as 'family'? Did you think of considering 'type of family' nuclear/ joint/ extended as a variable that may influence health care seeking? How can you best deal with the heteogenity of the small sample across all the variables when you wan to draw inferences based on the results of this qualitative study?

Response:

- The NMM and NMWs who consented to participate in the interviews were selected. The NMWs and NMMs who are actual residents of the villages and not migrants were selected.
- Data saturation led to the decision of numbers 10 and 7
- The NMW and NMM interviewed were not couples. Couples were not taken to avoid post-interview backlash, and the same decision with regard to family members.
- Family members were father-in-law and mother-in-law.
- The study did not consider the type of family as a selection criterion
- It is an exploratory study design and hence not conclusive, not policy driven but for strengthening MNCH interventions. The data was also triangulated from other stakeholders. Analysis of data from other stakeholders whether community members or health care providers, grounded the data from NMWs.

Change in the manuscript: Data saturation guided the decisions for the sample.

Page no. 6; line no. 133-134

6. **Query:** Was any of these characteristics related to the later findings? Eg religion with perceptions regarding preconception care, type of marriage with decision-making etc?

Response: The characteristics listed were from the inferences of the study. Deeper analysis and triangulation enabled us to understand these factors.

7. **Query:** Were the NMW from the sample already pregnant?

Response: The NMW inclusion criteria were – within one year of marriage. When found that NMW is pregnant she was excluded from the sample.

Change in manuscript: This sentence has been deleted

8. **Query:** This seems to be a general paragraph and not specifically about the result of this study?

Response: It is from the data, due to increased irrigation and demand for labour, more and more women are going to work as daily wage labourers. This has been corroborated by the responses of the participants.

Change in manuscript: This has been deleted. The second sentence has been revised as below:

The stakeholders pointed out that although women work in agricultural fields, their household responsibilities have not necessarily diminished, as they are still expected to manage housework such as cooking and caring for family members.

9. **Query:** Was this one of the variables studied in the study sample? Not mentioned in the characteristics table?

Response: Family members i.e., mother-in-law and father-in-laws are mentioned in characteristic table

10. **Query:** Only this is the data about work pressure? The current quote does not really support the 'work pressure', it does not specify what the work is.

Change in manuscript:

They have cookers now, anna, byali they will cook in the cooker and leave, they go to work in the field. Till night also if they eat anna byali only what they will get, Government easily gives rice, byali is available, they easily prepare… There is also pressure on them, to go, to work and earn, more wages are there now, want to go so they find easy thing. (MORD)

Page no. 10; line no. 209-212

…women go to work in the morning at 8.30 or so and don't come home till 7.00 in the evening. See since this irrigation started, it is like this (AWWRD)

Page no. 10; line no. 218-219

in the fields, only they work. After 10.00 AM, no one is available in the village at all… NMW also work, no one will be there in the house. They go… Even after delivery, they don't stay back for a month also… In some houses, if they go in the morning, they will come back in the evening at 6.00 PM. They carry a lunch box with them and go, they take it for the afternoon. (PHCORD)

Page no. 11; line no. 220-224

11. **Query:** Any specific finding to present about lack of decision-making powers affecting 'preconception care'?

Response: The data only provide information about the decision-making around the first conception and food purchase which can affect the health and nutrition during the preconception period.

Change in manuscript:

I will only decide about the gap, I am the husband, I will only decide about that, my wife will not decide about that. (NMM04YS, NMM)

The decisions around the purchase of food items are taken by the husband or the in-laws and the newly married woman is not involved in the decision-making process. This can affect the intake of nutritious food by the newly married woman.

vegetables and all my mother-in-law will only bring, elders in the family will only bring. May be my father-in-law or mother-in-law will bring it, not me. Elders whoever will be free will do all those things. (NMW04RD, NMW)

Page no. 11; line no. 232-239

12. **Query:** What are the perceptions of women vs men vs family members about preconceptions? What questions were asked to them to learn their perceptions? What do they think as preconception care?

Response: The concept of preconception care is novel for the community. So both are equally unaware of the need. However, the responses of NMWs reflected that this could be accepted by women. Also when explained and asked about PCC, NMWs were more open to the idea.

The question asked is as below:

Can you tell us about your thoughts on preparation for pregnancy keeping in mind the future baby to be born? How should your health be? (Probe: have you heard about such things in your family/ community, traditionally any norms and taboos are followed? Tobacco/supari/alcohol/smoking)

13. **Query:** The sample size is small- hence suggest to avoid these terms- some vs many

Response: Rectified

14. **Query:** The previous sentence and this sentence- please re phrase to clarify- whether they had info or they didn't.

Response: Rearranged entire section

Change in manuscript:

The concept of preconception care is novel for the community. The stakeholders pointed to the fact that there are no interventions currently being implemented that target women prior to conception and all the existing interventions focus on women once she conceives:

> *…we don't have that program (newly married couples) …But I don't have an idea about that. Only after pregnancy we enrol, after delivery, we enrol those who have anyone come from outside. (CDPORD)*

> *There is no scope for that (including newly married in VHND and meetings for pregnant women), that category is not there, they have not been included in any of these things…. We are also neglecting them, where there are injections for them? where there is a provision to call them? after they become pregnant, we start check-ups for them. there is no program to involve them in between. (CDPORD)*

> *I don't know about this, I don't have information about this (NMM04YS, NMM)*

As part of the study, we tried to understand the views around introducing preconception care services from all the stakeholders. Many, although not all, of the stakeholders, revealed their stance towards the idea of strengthening and expanding programs to improve women's nutrition at the preconception stage. The Family and Community Members group was almost evenly split between those who overtly voiced support for intervening further upstream, versus those who either did not speak to this topic or voiced pessimism about such an approach. Three out of the seven NMMs had a positive opinion, and seven out of 10 of the NMWs were supportive of preconception care programs. The responses have been mentioned below:

Yes, it is necessary that she has to take care of it… No, we have not done any preparations. (NMM07YS, NMM).

Information should be given in the school and college…. if a demonstration is given it will be good, it will be helpful. (NMW06YS, NMW)

There is no need to bring (anything and give) before pregnancy. (NMM06YS, NMM)

The public healthcare providers and policymakers expressed support for preconception nutrition efforts. The District Officials group was generally supportive of preconception nutrition awareness-raising, with only one-third not voicing support.

Page no. 13-14; line no. 271-298

The recommendations from government officials and policy implementers highlighted the need for such interventions in improving maternal and child health outcomes:

We are doing it for pregnant women, we can do it for them also (newly married women). Then they will look after correctly…we are doing so much work, along with that we can also tell a few things to the newly married women. (AWWRD)

…reaching her before (pregnancy) is important because, if she knows all the information, if she gets all the information, then, in future when she becomes pregnant, she will know how she should be and how she should eat, all this information she will come to know, she will tell her family about that also. (AWWRD)

Much difference of opinion was obtained from stakeholders for the appropriate timing for targeted awareness-raising and nutrition or health interventions. Stakeholders fell into one of three camps: those that advocated for intervening only after pregnancy, those who preferred targeting all newly married women, and those who believe that waiting till marriage to raise awareness is too late.

That we can think after becoming pregnant. (NMW01RD, NMW)

…to get that outcome or prevent anaemic pregnancy or maternal death, infant death all those things. So we have to concentrate more on adolescents (MORD)

Stakeholders mentioned that eligible couple listing is a routine exercise by ASHAs and there is an opportunity to integrate interventions identified exclusively for the couples before pregnancy along with family counselling:

The eligible couples (EC) listing is happening continuously. (MORD)

The data from our qualitative research emphasised that leveraging key services and programs in RMNCH+A could act as a prime facilitator for preconception care.

Page no. 15-16; line no. 327-358

15. **Query:** Were the NMW NMM and family members asked about whether, where and how they would like to receive the preconception care?

Response: Yes, and also the other stakeholders like academicians, health care providers and implementors were asked about preconception care interventions.

The need for PCC is strong by stakeholders; PCC interventions are not defined and hence where and how has not been answered well.

When discussed/probed about PCC components stakeholders referred to ASHA and AWW to seek such care/services

"It may not be possible to gather everyone together but awareness needs to be given. it can be done through anganwadi or ASHA worker or women groups can be created to give awareness and knowledge about nutrition". (NMW04RD, NMW).

Change in manuscript: Leveraging existing programs

Page no. 17; line no. 367

16. **Query:** These specific programs are mentioned in the table and in the narrative the category of interventions are detailed.

For Ex: nutrition-related interventions include ICDS, Mathrupoorna etc.

Changes in manuscript: Deleted lines 376-419

Rearranged the section:

Nutrition-related interventions

Iron and folic acid tablets are provided to pregnant women after registration and the opinion of a few stakeholders was to initiate the supplements before a woman conceives to help her have good stores before she conceives:

…Provide nutrition supplementation for those who need it. Expand RBSK / RKSK programs to include these components. (SNO).

…Then, tablets and all, calcium tablets, they can't buy, so the government gives calcium tablets. Whatever they need those should be supplied. (NMWYS07, NMW)

The provision of food in the form of hot cooked meals was strongly recommended to bridge the gap in nutrient intake. Stakeholders thought to extend the provision of hot cooked meals as part of the Matrupoorna Yojane by the government of Karnataka which is presently provided to pregnant women to undernourished women before conception:

They should give them meal there only because they give ration and all, and if they give it home, I don't know if they will get it (use it for themselves or not) or not. They should call them there, give them food there and tell them, eat egg and all like this… they prepare it for the children and give (NMWRD01, NMW)

…They should give nutrition food to those whose nutritional status is not good, like they give eggs to pregnant women, like that they should give it would be best. (NMWYS06, NMW)

…There should be some service to give them some nutrition or something after marriage may be for three to six months… may be some powder for six months after marriage… After that, if we want, we can continue. Or we can just complete six months course and if they are pregnant, we can continue with other things. (MLHP01YS)

Education and Awareness-raising

There was greater unanimity on this theme than any other; every stakeholder group repeatedly emphasized the need to better educate the public regarding nutrition. A few stakeholders recommended a robust awareness-raising campaign:

A concerted communications/education campaign needs to be rolled out to ingrain the importance of nutrition and raise awareness of things like haemoglobin. (SNO)

Other interventions proposed included expanding nutrition education in schools:

Nutrition education in school should be improved/expanded. Starting in 4th standard, kids should be taught about healthy dietary habits, vitamin deficiencies and diet-related illnesses. Currently, private schools teach this but not public-sector schools. (SNO)

Others suggested educating parents in particular:

Parents should have the knowledge my daughter is growing and she needs more nutrition and extra education about nutrition for future needs. So those kinds of things are there. knowledge level should improve then everything will improve. Especially from the school level we should start explaining, that is very much needed. By the time she comes to a doctor, she will be anaemic. She will be anaemic, she will be malnourished, and she will be underweight. So, if we start from basic it will be good. (RCHOYS)

Besides education at the family level, the Healthcare Providers (HCPs) should provide adequate messaging and information about the services provided to the beneficiaries and their families for effective utilization and impact.

For example, there is room for improvement in ASHA workers' messaging and counselling, when instructing pregnant women on the proper use of supplements:

Page no. 19-21; line no. 420-469

17. **Query:** Who is SNO?

Response: State Nodal Officer for ASHA programs

18. **Query:** So is this ‘leveraging existing intervention or new intervention?’

Response: It is under leveraging existing interventions and has now been moved to the respective section

19. **Query:** Is this about pregnant women or adolescents? How is it under ‘new interventions?’

Response: The reference is to pregnant women

Change in manuscript: Moved to leveraging section

Page no. 19-21; line no 420-469

20. **Query**: Is something missing after this?

Response: This is a mistake; deleted

Change in manuscript: Deleted: Page no. 22; line no. 504-507

21. **Query**: So is this ‘leveraging existing intervention or new intervention?’

Response: Education, awareness and nutrition-related interventions are moved to leveraging section and only screening is retained in the new interventions

Change in manuscript: Rearranged section: Page no. 19-21; line no. 420-469

22. **Query:** Have you not inlucded the Matrupoorna yojna to be extended to undernourished women, in the following table?

Response: It has been included now

The table below summarises the interventions which include **leveraging existing programs and new interventions** during the preconception period:

Table 1: Summary of the interventions during the preconception period

**Leveraging and strengthening existing programs to include care during the preconception period**	
**Existing programs**	**Inclusion**
**Poshan Abhiyaan Program**[13]	Poshan Abhiyaan can be extended to provide nutrition education and reproductive health counselling before pregnancy through:<br>1. Counselling initiatives for newly married couples and awareness for couples and families through Primary Health Centre teams (including Health and Wellness Centres) and frontline workers<br>2. Awareness programs for other key community stakeholders such as Gram Panchayat members and religious leaders<br>3. Awareness programs for adolescents on preconception care, which can be a multi-department effort
**Village Health, Sanitation and Nutrition Committee (VHSNC) and Village Health and Nutrition Day (VHND)**	VHSNC and VHND meetings can be leveraged for preconception care discussions

**Pradhan Mantri Surakshit Matritva Abhiyan (PMSMA)**[14]	The PMSMA camps, which are scheduled every month, can include a preconception care concept
**Anaemia Mukt Bharat program under the National Health Mission**[15]	Micronutrient supplementation of iron and folic acid provided as part of the anemia mukt Bharat program for women of reproductive age can be ensured for women planning to conceive
Mathrupoorna **Scheme**[16]	The hot cooked meal provided to pregnant and lactating women through the existing system of Anganwadi Centers as part of the Mathrupoorna Scheme can be extended to undernourished women planning to conceive.
**Potential new interventions targeting preconception nutrition care planning**	
**New interventions**	**Activity**
**Screening & Management**	1. Calculating Body Mass Index (BMI)<br>2. Hb estimation for anaemia prevention and treatment<br>3. Testing for diabetes, thyroid disorders, hypertension other biochemical estimations<br>4. Regular follow-up - health check-ups for weight and underlying condition management

Page no. 24- 27; line no. 546-548

23. **Query:** Even the previous sub section inlucded some part of 'how' along with 'what'.

Response: The how part is moved here with few edits in track change mode

Change in manuscript: The secondary and tertiary stakeholders have suggested how preconception care services can be provided to newly married couples. They include:

- Involving the gram panchayat to deliver preconception care services

The predominant strategy to deliver preconception care services suggested by stakeholders was to involve the whole panchayat in counselling newly married couples at the community level:

In this season (marriage season, February-May) we can have these awareness programs, we can invite them and like this… what we should do is, at the panchayat level, we should call the newly married couple and we can give them training. (EOYS)

Involving the Gram Panchayat is the way forward to ensure good health and nutrition of NMW, as suggested below:

At the panchayat or PHC level, kitchen garden or other diet-focused interventions should be rolled out. (MORD)

"…what we should do is, at the panchayat level, we should call the newly married couple and we can give them training… If we hand this over to the village health committee, it will be at the village level and it will not be a burden. I suggested gram panchayat, but because there is this team at the village level, they need not come to the village panchayat level also." - (EOYS)

"…as PDO I can bring together the community and other departments. Gram panchayat is there to bring all departments together and carry on the activities. So, in that direction as PDO I can bring together people and whatever committees are there, I can support them to achieve the objectives and also deal with malnutrition. Like that, we should take precautions. Before pregnancy, if we take precautions then if there is any health issue like thyroid, it can easily be managed." - (PDOYS)

Skill development courses for adolescent girls at the panchayat level were suggested for life skill development about food, cooking, nutrition, and family management.

- Engaging the husband and family members of the newly married women

Another strategy suggested was the engagement of men and family members in decisions around the health and nutrition of the woman. Participants of the study emphasized educating various members of the household on the importance of not only nutrition but generally caring for and ensuring the health of newly married women.

"… also, elders should be given this information, they will also help. Parents will be there in that house, mother-in-law, father-in-law, they only look after us, isn't it? So, they should be given information. in meetings also they can tell, and they can tell during house visits also. " - (NMW04RD, NMW)

Educating men and creating awareness on nutrition and birth spacing and making them aware that women's health should be of high priority as suggested below:

"Main role should be men, we should give awareness to men only, and we should say, see these women have come trusting you… She gives birth, you should care about her, show concern for her, you should feed her, and show affection. We should tell them; we should tell them only." - (PDORD)

Numerous responses from NMM like the one below made clear that such engagement has been lacking, and that healthcare providers and educators have failed to reach out to them with relevant information and guidance.

"No one has spoken about this with me. No one has told me about this. No information, no one has told. I have not spoken to anyone about this" - (NMM02RD, NMM).

- Convergence and collaboration across different government departments

  Strengthening intra- and inter-governmental coordination, both laterally (across departments) and vertically (national, state, and district levels) was suggested by stakeholders for the effective implementation of preconception care programs. There is an inclination towards collaborative efforts rather than ownership by a single department:

Mainly school, education department, anganwadi, health, Integrated Child Development Services (ICDS) and health. Three of them should combine. (MORD)

- Clear articulation of respective roles and responsibilities of health cadres for the provision of preconception care services:

"For successful implementation, the three 'A's need to be involved: ASHAs, Auxiliary Nurse Midwife (ANM), and Anganwadi. ASHAs will be mobilizers, ANMs will be health providers, and Anganwadi will be nutrition advisors. In case communities resist the three 'A's (ASHA, anganwadi worker (AWW) and auxiliary nurse midwife (ANM)), village leadership should be engaged." (SNO)

- Encouraging greater autonomy and leeway for customization of preconception care interventions at the district level (using Needs Assessments):

"Each district has its unique context, and they should be able to determine which specific age range should fall in the "preconception" period. The pubertal period is critical for nutrition as that's when the growth spurt happens." (Academician)

Community Needs Assessment (CNA) can help understand specific needs. It is being re-started in the context of Comprehensive Primary Healthcare (CPHC). (Academician)

National and state levels need to be very involved at the planning stage but districts need to be given enough autonomy to innovate and develop their own tailored approaches. Expectations around data reporting need to be reasonable so as not to overly burden the implementation process. (Academician)

Page no. 27-30; line no. -550-651

24. Query: Was it only less educated? Did the results point this out?

Response: Corrected

Change in manuscript: The stakeholder interviews and discussions revealed that the concept of preconception care generally resonated and was supported by all government officials and healthcare providers, but few of the primary stakeholders questioned the need and were of the mindset that no type of preparation for pregnancy is necessary. One key disagreement point among many stakeholders was around the appropriate timing for introducing

Page no. 32; line no. 664-667

25. **Query:** I am not sure whether this has clearly come in the results, please ensure.

Response: Added in the results (Line 344-352; page no. 16)

Change in manuscript: One key disagreement point among many stakeholders was around the appropriate timing for introducing preconception care interventions. (Line 668-669; page no. 32)

26. **Query:** Suggest to add a reference regarding uptake of specifically the preconception care and not just generally about maternity services.

Response: Our search did not yield any references about socio-cultural factors and preconception care service uptake. Please suggest if we can remove this reference?

27. **Query:** As new interventions?

Response: We are listing only the intervention domains and not specifically mentioning new one existing; hope it is fine now.

Change in manuscript: The interventions can be categorized into the following domains: education and awareness, nutrition-related interventions, health and nutrition screening with monitoring and family-focused counselling. (Line 695-697; page no. 33)

Additional changes in manuscript

1. The qualitative study findings provide a basket of interventions suggested by government functionaries which include leveraging existing programs and new interventions during the preconception period targeted at women of reproductive age.

   Page no. 3; line no. 52-54

2. The qualitative data analysis software NVivo was used for documentation and organizing data into themes and sub-themes.

   Page no. 6; line no. 129

3. The healthcare providers and government functionaries, consistently raised stress as a serious health risk facing newly married women—one that has real physical manifestations that may intersect with dietary behaviours as well as general maternal health and birth outcomes.

   Page no. 8; line no. 168

4. Similar thoughts were also shared by another stakeholder:

   Page no. 9; line no. 182-183

5. Often it is mentioned that the newly married feel scared to speak their mind to their mother-in-law or father-in-law, as the healthcare provider says:

   Page no. 12; line no. 257-258

6. List of abbreviations:

   Page no. 33; line no. 723-727

   Services; IDI: In-depth Interview; KHPT: Karnataka Health Promotion Trust; NMM: Newly Married Men; NMW: Newly Married Women; RBSK: Rashtriya Bal Swasthya Karyakram; RKSK: Rashtriya Kishor Swasthya Karyakram; RMNCH+A: Reproductive, Maternal, Newborn, Child & Adolescent Health; PHCO: Primary Health Care Officer; VHSNC: Village Health Sanitation and Nutrition Committee: VHND: Village Health Sanitation and Nutrition Day

7. The study was conducted between September 2021 through January 2022. T

   Page no. 5; line no. 110-111

8. Table 1 title

Table 2: Characteristics of the participants (primary stakeholders) interviewed in taluks Shorapur and Devadurga **between September 2021-January 2022**

Page no. 6; Line no. 142-143

9. Table 2 title

Table 3: Summary of the suggested interventions during the preconception period by different stakeholder groups who were interviewed between September 2021-January 2022

Page no.24; Line no. 550-551
